# Prevalence of SARS-CoV-2 in Communities Through Wastewater Surveillance—a Potential Approach for Estimation of Disease Burden

**DOI:** 10.1007/s40726-021-00178-4

**Published:** 2021-04-06

**Authors:** Prosun Bhattacharya, Manish Kumar, Md. Tahmidul Islam, Rehnuma Haque, Sudip Chakraborty, Arslan Ahmad, Nabeel Khan Niazi, Zeynep Cetecioglu, David Nilsson, Julian Ijumulana, Tom van der Voorn, Md. Jakariya, Maqsud Hossain, Firoz Ahmed, Mahbubur Rahman, Nargis Akter, Dara Johnston, Kazi Matin Ahmed

**Affiliations:** 1 Department of Sustainable Development, Environmental Science and Engineering, KTH Royal Institute of Technology, Teknikringen 10B, SE-11428 Stockholm, Sweden; 2 KWR Watercycle Research Institute, Groningenhaven 7, 3433 PE Nieuwegein, The Netherlands; 3 Discipline of Earth Science, Indian Institute of Technology Gandhinagar, Ahmedabad, Gujarat 382 355 India; 4 WaterAid Bangladesh, House 97/B, Road 25, Block A, Banani, Dhaka, 1213 Bangladesh; 5 Environmental Interventions Unit, Infectious Disease Division, icddr,b, 68 Shaheed Tajuddin Ahmed Sarani, Mohakhali, Dhaka, 1212 Bangladesh; 6 Department of DIMES, University of Calabria, Via P. Bucci, Cubo 42/a, 87036 Rende, Italy; 7 Department of Environmental Technology, Wageningen University and Research (WUR), Droevendaalsesteeg 4, 6708 PB Wageningen, The Netherlands; 8 Institute of Soil and Environmental Sciences, University of Agriculture Faisalabad, Faisalabad, 38040 Pakistan; 9 School of Civil Engineering and Surveying, University of Southern Queensland, Toowoomba, Queensland 4350 Australia; 10 Department of Chemical Engineering, KTH Royal Institute of Technology, Teknikringen 42, Stockholm, SE-10044 Sweden; 11 WaterCenter@KTH, Department of Sustainable Development, Environmental Science and Engineering, KTH Royal Institute of Technology, Teknikringen 10B, SE-11428 Stockholm, Sweden; 12 University of Osnabrück, Institute of Environmental Systems Research, Barbarastr. 12, 49069 Osnabrück, Germany; 13 Department of Environmental Science and Management, North South University, Bashundhara, Dhaka, 1229 Bangladesh; 14 Department of Biochemistry and Microbiology, NSU Genome Research Institute (NGRI), North South University, Bashundhara, Dhaka, 1229 Bangladesh; 15 Department of Microbiology, Noakhali University of Science and Technology, Noakhali, Bangladesh; 16 Water Sanitation and Hygiene Section, United Nations Children’s Fund, BSL Office Complex, 1 Minto Road, Dhaka, 1000 Bangladesh; 17 Department of Geology, University of Dhaka, Dhaka, 1000 Bangladesh

**Keywords:** COVID-19, SARS-CoV-2, Prevalence, Wastewater surveillance, Monitoring, Developing Countries

## Abstract

The episodic outbreak of COVID-19 due to SARS-CoV-2 is severely affecting the economy, and the global count of infected patients is increasing. The actual number of patients had been underestimated due to limited facilities for testing as well as asymptomatic nature of the expression of COVID-19 on individual basis. Tragically, for emerging economies with high population density, the situation has been more complex due to insufficient testing facilities for diagnosis of the disease. However, the recent reports about persistent shedding of viral RNA of SARS-CoV-2 in the human feces have created a possibility to track the prevalence and trends of the disease in communities, known as wastewater-based epidemiology (WBE). In this article, we highlight the current limitations and future prospects for WBE to manage pandemics.

## Introduction

Severe acute respiratory syndrome coronavirus 2 (SARS-CoV-2) has been identified as the cause of ongoing pandemic of severe pneumonia known as COVID-19. The first COVID-19 cases were reported from China in December 2019, followed by a rapid spread to other countries in a matter of a few weeks. The World Health Organization (WHO) declared the status of this outbreak from epidemic to pandemic on March 11, 2020 [1]. The first confirmed clinically diagnosed case of COVID-19 in the EU was announced on January 24, 2020, and the corona cases have been increasing dramatically in countries across all continents [2].

The currently reported figures are primarily based on patients who have been clinically tested. The asymptomatic nature of COVID-19 among several population cohorts leads to considerable uncertainty in estimation of the actual number of patients and the true situation of COVID-19 spread [1, 3, 4••, 5•]. It is therefore difficult to quickly determine the extent of COVID-19 spread within a population. In emerging economies with high population density such as India, Bangladesh, Pakistan, Brazil, Sri Lanka, and others, the situation is complex, as a significant number of SARS-CoV-2 affected patients are likely to be asymptomatic and oligosymptomatic, which are generally not clinically tested and therefore create uncertainty in the estimation of the disease burden of COVID-19 [6, 7]. First confirmed cases of SARS-CoV-2 RNA in municipal wastewater were reported from Netherlands [8••], Australia [9•], Japan [10•], India [11•], Italy [12•], Spain [13•], Sweden [14•], Bangladesh [15••], and the USA [16, 17•]. These findings were also concomitant to the first officially confirmed clinically diagnosed cases in these countries. These findings could act as a proven concept for potential, although hidden, information on the prevalence of SARS-CoV-2 infections and offer a cost-effective alternative to screen a large number of random individuals in the population. Researchers from Spain identified the genetic material of SARS-CoV-2 in preserved wastewater samples from March 2019 while it was not clinically diagnosed [18•]. Several studies have indicated a close relationship between the fluxes of human viruses in wastewater as well as surface water with the health status of communities [19•, 20•]. The concentration of viruses in both wastewater and surface water bodies could serve as a proxy for monitoring of the circulation of human viruses among the population [7, 16, 18•, 19•, 20•, 21]. Recently, some reports have identified that infection with SARS-CoV-2 is accompanied by persistent shedding of viral RNA in the human feces in 27 to 89% of COVID-19 infected patients at densities from 0.8 to 7.5 log_10_ gene copies per gram [18•, 22]. In contrast to a long-way process of laboratory testing of individual patients, the presence of SARS-CoV-2 RNA in feces enriches to sewage water. This creates a promising opportunity to investigate the genetic constituents of SARS-CoV-2 in wastewater [23••]. This approach is referred as the wastewater-based epidemiology (WBE) and could be used for surveillance of the enteric viruses in environmental matrices [5•, 8••, 9•, 10•, 12•, 13•, 14•, 15••, 16, 17•, 23, 24••, 25–28].

## Risk From Wastewater Spillage in Developing Countries

Around 20% people are still open defecating in the Central and Southern Asia and and Sub-Saharan Africa regions, and on the other hand, in the Sub-Saharan Africa region, only 20% of the total population have improved hygienic sanitation facilities [29–31]. In the Central and Southern Asia region, only 40% of the urban population have improved hygienic facilities whereas the hygienic sanitation facilities are almost absent. As a result, the transfer of human feces enriched wastewater from non-sewered sources to unplanned discharge places, in most cases, pose a potential threat for contamination of the surface water sources, [24••, 32••, 33•]. It is important to mention that in several developing countries, a significant proportion of population are dependent on groundwater sources for drinking purpose without adequate purification and thus in a potentail rsk of exposure to different types of contaminants. In this particular context, it is imperative to look at the possible of the presence of any virus as indicated in different literature in the past concerning the presence of viruses in groundwater [34, 35], including the genetic materials of SARS-CoV-2 virus in the groundwater system [32••].

**Fig. 1:**
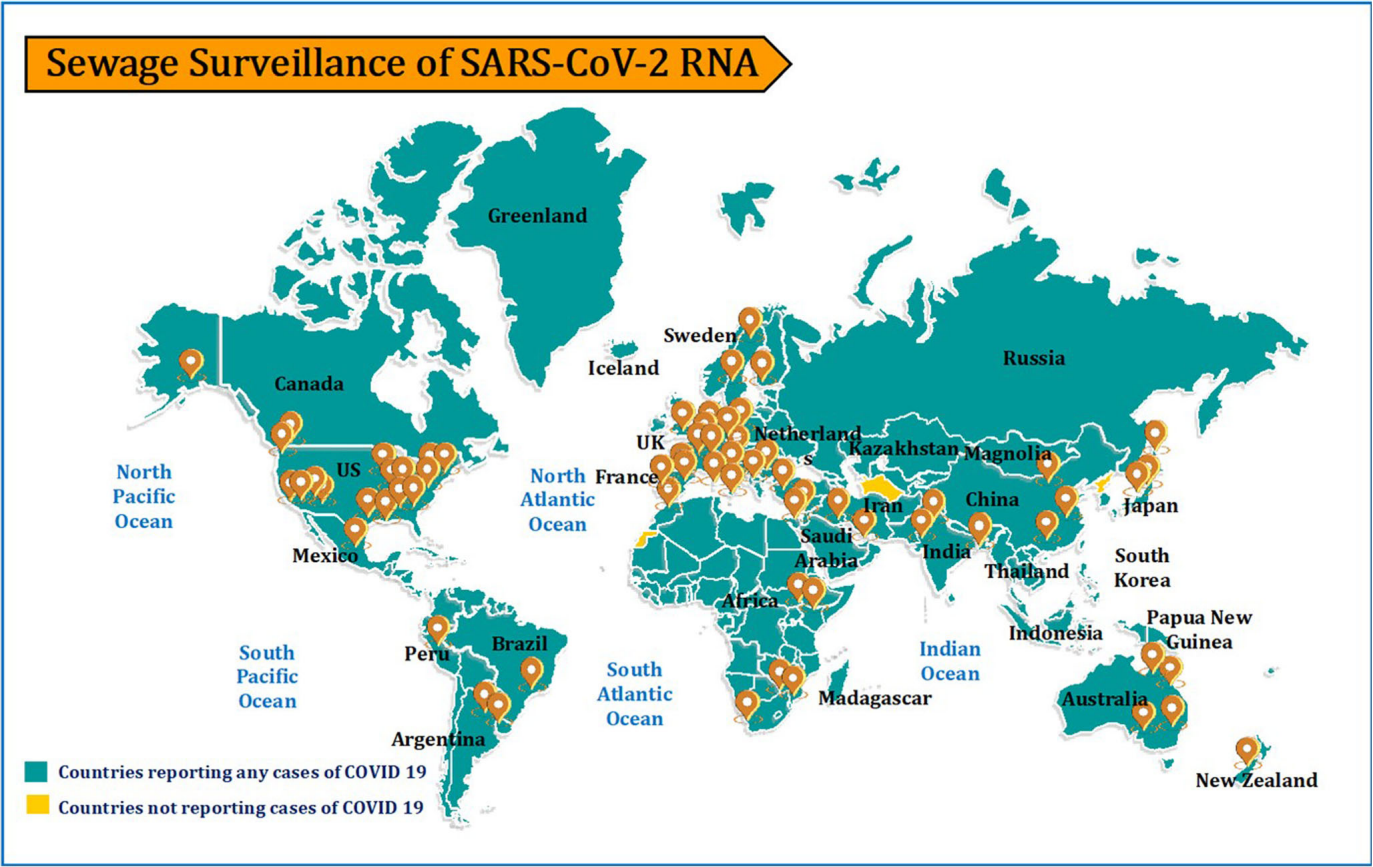
Location of institutions in the world, including the emerging economies, conducting sewage surveillance for early detection of SARS-CoV-2 outbreak (adopted from CDC—https://www.cdc.gov/coronavirus/2019-ncov/global-covid-19/world-map.html)

The detection of the SARS-CoV-2 virus via RNA in environmental waters has so far not been proven as a potential pathway transmission of COVID-19, and still, there is a need for international and national efforts for assessing the risk from SARS CoV-2 being spread through water [34, 35]. Studies carried out over the past decades have indicated that viruses exhibit very different survivability characteristics, and the surrogates may not accurately predict the environmental fate of the pathogenic virus. Quantitative risk assessments should be conducted for highly pathogenic enveloped viruses in wastewater, recreational waters, and drinking waters, and also in the groundwater. In order to develop both capacity and proper institutional setup in different countries worldwide, national sewage and wastewater authorities should be encouraged to develop the capacity as well as tools to monitor wastewater (Fig. 1). Despite the growing volume of literature on WBE, little is yet known about how COVID-19 spreads or travels through groundwater resources, not to mention its potential repercussions for groundwater management [33•, 36]. Considering the current challenges for using wastewater as a tool for the surveillance of the SARS-CoV-2 are to a major extent due to analytical methods, and more specifically related to:

- RNA isolation step: the most limited and less known step is the isolation of nucleic acid. Therefore, this step should be improved resulting in more cost-effective, and coverage of the method should be calculated with standards [14, 37, 38].
- Recovery efficiency during isolation of RNA: there are different approaches to concentrate viruses before RNA isolation step; however, their recovery efficiency needs to be investigated [14, 37, 38].
- The estimation tool linking the population data and sewage data; modeling for an outbreak and sewage estimation should be linked.
- The microbiology of the built environment: improving our understanding of viral communities in the built environment is needed, specifically which viruses are present and their sources, spatial and temporal dynamics, and interactions with the bacterial population [39].

## What Are the Potential Role and Limitations of WBE Applications?

WBE application provides an opportunity to monitor the trend of SARS-CoV-2 spread in communities especially in areas with non-sewered sanitation, through developing a systematic approach, which includes identification of appropriate locations, sampling procedures, preservations from the sites to the laboratory, isolation of the SARS-CoV-2 genetic materials, and recovery. In order to achieve an optimal result from WBE applications, it is important to detect, quantify, and analyze the trends in the spatial and temporal abundance and variability of the genetic materials of the SARS-CoV-2 in environmental water. Some important factors which need to be considered are:

- Developing standard protocol for sample collection and robust methods for extraction of the viral RNA and analysis of the target genetic materials
- Physiochemical characteristics of the water such as Chemical Oxygen Demand (COD), Total Kjelldahl Nitrogen (TKN), Total Dissolved Solids (TDS), and Total Suspended Solids (TSS) used to calculate the served population which is strictly connected to human feces which could improve the accurate estimation; and
- Demographic details and actual data of the clinically diagnosed COVID-19 patients in a particular area or locality necessary to analyze the consistency of the results for the communities.

WBE surveillance in sewage complement the current clinical surveillance, on patients with SARS-CoV-2, demonstrated that the increase of the average viral load of SARS-CoV-2 in wastewater samples with respect to time precisely followed the increase in the number of fatal cases of SARS-CoV-2. WBE can be successfully applied only by data sharing and coordination of methodologies among different country and society.

The presence of SARS-CoV-2 in wastewater is detected before the beginning of the exponential growth of the epidemic [3]. Same aspects have been observed experimentally with the circulation of SARS-CoV-2 in wastewater even before the first cases were reported by authorities [13•]. Thus, a simple theoretical approach of WBE methodology starts from the concentration of SARS-CoV-2 (converted into gene copies/m^3^) measured in municipal wastewater taken along the network or at the inlet of a WWTP, but at a point that represents a known urbanized area drained by the sewer system. The daily viral load in wastewater (expressed in copies/d) is then calculated. Unfortunately, analytical data later confirmed that the load of SARS-CoV-2 virus in feces is highly variable. Despite of all these challenges, detection and monitoring of genetic material of SARS-CoV-2 in wastewater could be deployed as a system for surveillance of the prevalence of the disease burden in the communities [40••].

In summary, WBE could be a promising tool for SARS-CoV-2 surveillance in wastewater, but extensive and highly coordinated studies are required, including the quantification of individual virus load in feces and during the disease, because this information is very uncertain at the moment but is fundamental for accurate estimations. It is also important to mention that the origin of many natural resource management problems, particularly water and sanitation challenges in developing countries, are related to the governance of the service delivery provisions, which also pose risk for increased vulnerability to COVID-19, lacking adequate mechanisms for governing the COVID-19 crisis [41, 42].

## Way Forward

Several scientific pieces of literature on the occurrence of contaminants originating from pit latrines and the factors affecting the transport of these contaminants conclude with the note that they may negatively affect human health. Sewage can directly enter natural water systems due to lack of wastewater treatment plant, overflow, or complete lack of the sewer network providing a pathway for groundwater contamination. Hydrogeological conditions for any particular area play a key role in setting the safe distance from drinking water sources like tube well to pit latrine, and accordingly, the horizontal distances and depth vary from site to site [4••, 41, 43, 44, 45••]. Therefore, WBE tool can provide a new horizon of learning about the characteristics of novel viruses like SARS-CoV-2 and its presence of the genetic materials in groundwater in the developing countries while implementing the provision of safe drinking water service delivery, from groundwater sources without any prior treatment.

Development of an international platform for research collaboration can bring quick and positive result where every individual community or country would be encouraged to contribute depending on their ability and more importantly by sharing each other’s knowledge and expertise. At the same time, considering the urgency and the nature of the spread of the SARS-CoV-2 and other similar types of viruses, it is imperative to develop a rapid and economic WBE tool for monitoring the status and trends of COVID-19 mass infection level despite the current challenges faced by the pioneering countries in this particular context. Some of these challenges include a statistically representative sampling of sewage, size of the population, and the volume of sewage water. Considering the existing situation and the availability of technical knowledge and financial limitation, the global community needs to develop a WBE tool to detect and monitor the spread of COVID-19 without further delay.
